# RNAseq expression patterns of canine invasive urothelial carcinoma reveal two distinct tumor clusters and shared regions of dysregulation with human bladder tumors

**DOI:** 10.1186/s12885-020-06737-0

**Published:** 2020-03-24

**Authors:** Heidi G. Parker, Deepika Dhawan, Alex C. Harris, Jose A. Ramos-Vara, Brian W. Davis, Deborah W. Knapp, Elaine A. Ostrander

**Affiliations:** 1 National Human Genome Research Institute, National Institutes of Health, 50 South Drive, Bldg 50, Room 5351, Bethesda, MD 20892 USA; 2 Department of Veterinary Clinical Sciences, College of Veterinary Medicine, Purdue University, West Lafayette, IN 47907 USA; 3 Department of Comparative Pathobiology, Purdue University, West Lafayette, IN 47907 USA; 4 Department of Integrative Biological Sciences, Texas A and M University, College Station, TX 77840 USA; 5 Purdue University Center for Cancer Research, Purdue University, West Lafayette, IN 47907 USA

## Abstract

**Background:**

Invasive urothelial carcinoma (iUC) is highly similar between dogs and humans in terms of pathologic presentation, molecular subtypes, response to treatment and age at onset. Thus, the dog is an established and relevant model for testing and development of targeted drugs benefiting both canine and human patients. We sought to identify gene expression patterns associated with two primary types of canine iUC tumors: those that express a common somatic mutation in the *BRAF* gene, and those that do not.

**Methods:**

We performed RNAseq on tumor and normal tissues from pet dogs. Analysis of differential expression and clustering, and positional and individual expression was used to develop gene set enrichment profiles distinguishing iUC tumors with and without BRAFV595E mutations, as well as genomic regions harboring excessive numbers of dysregulated genes.

**Results:**

We identified two expression clusters that are defined by the presence/absence of a BRAFV595E (BRAFV600E in humans) somatic mutation. BRAFV595E tumors shared significantly more dysregulated genes than BRAF wild-type tumors, and vice versa, with 398 genes differentiating the two clusters. Key genes fall into clades of limited function: tissue development, cell cycle regulation, immune response, and membrane transport. The genomic site with highest number of dysregulated genes overall lies in a locus corresponding to human chromosome 8q24, a region frequently amplified in human urothelial cancers.

**Conclusions:**

These data identify critical sets of genes that are differently regulated in association with an activating mutation in the MAPK/ERK pathway in canine iUC tumors. The experiments also highlight the value of the canine system in identifying expression patterns associated with a common, shared cancer.

## Background

Urothelial carcinoma is the second most common cancer of the urinary tract in humans following prostate cancer [1]. Approximately 80,000 new cases are diagnosed each year with 17,670 people expected to die from the disease this year [2]. The invasive form of urothelial carcinoma (iUC), which comprises 25–30% of human bladder cancer cases, is the most common urinary bladder tumor of dogs. It accounts for ≥90% of canine bladder tumors, with ≥50,000 dogs predicted to develop the disease yearly in the U.S. alone [3, 4]. In dogs, the tumors are naturally occurring, with the majority being high-grade papillary infiltrative tumors [3]. Distant metastases are present in about 15–20% of dogs at diagnosis, and in ≥50% of dogs at death, with liver, lung, and bone being frequent sites of metastases [3–6].

Invasive urothelial carcinoma is similar between dogs and humans in terms of pathologic presentation including cellular features and tumor heterogeneity, molecular subtypes (basal and luminal), and response to treatment [1, 4, 7]. The most common clinical signs at presentation in dogs include blood in the urine, frequent and painful urination and frequent small voids [7]. Diagnosis of iUC is made by histologic assessment of biopsies obtained via cystoscopy or surgery. While complete cystectomy is often a front-line treatment for human iUC, it is rarely used in dogs as tumor growth into the urethra is common at presentation, as is metastasis. The morbidity and cost associated with the procedure is a further deterrent for both surgeons and patient families. Instead, the most common treatment includes COX inhibitors and chemotherapy, offered together or separately [7, 8].

In the past several years canine iUC has been established as a relevant model for the testing and development of targeted drugs that benefit both canine and human patients [3, 9, 10].. Because of similarities in molecular features, tumor heterogeneity, metastatic behavior, and an immunocompetent host, the dog system closely mimics the human condition. It is, thus, expected that the canine system will predict drug outcomes in humans with high fidelity. Examples of targeted agents currently undergoing testing in dogs include folate-vinblastine, [11] folate-tubulysin, [12] and 5-azacitadine [13].

Genomic analyses of human iUC reveal multiple tumor subtypes, identified through expression profiles, often combined with somatic mutations and/or genomic rearrangements [14–21]. As few as two, and as many as nine, overlapping clusters of human iUC tumors have been identified, with results highly dependent on study design factors such as analysis methods, number of tumors analyzed and types of data collected [14–21]. Despite variation across studies, there is strong evidence that clustering patterns can predict disease progression and treatment response [14, 22–24]. This is due to the occurrence of some common clusters across studies including the division between luminal and basal-like tumors and the identification of expression-based clusters related to immune function that indicate infiltration of the tumors by immunologic cells including lymphocytes, macrophages, and myofibroblasts [16, 17, 20].

Two studies of gene expression in canine iUC, one based on expression arrays and another using RNAseq identified a similar basal/luminal division as that observed in human iUC cancers [25, 26]. Two additional canine iUC RNAseq studies, however, which included seven and 11 tumors, respectively, reported no distinct clusters, although both reported similarities between canine and human tumor expression profiles, including altered expression in genes or pathways involved in cell cycle progression, DNA repair, and inflammation [27, 28]. These differences indicate that there is much we still don’t understand about iUC specific expression profiles in dogs, and additional consideration is needed which uses a genome-wide, rigorous and unbiased approach to delineating regulation of genes critical to expression profiles, as we have done here.

Herein we have sequenced the transcriptomes of 15 canine iUC tumors and identified two expression clusters that can be defined by the presence or absence of a BRAFV595E somatic mutation which we have previously observed in > 85% of canine iUC tumors [29, 30]. The same mutation in humans (termed BRAFV600E) is present in several human cancer types including melanoma, colorectal cancer, thyroid cancer, and hairy-cell leukemia [31]. In this study we present the primary sources of differentiation between the two tumor clusters, both in terms of types of genes altered as well as regions of the genome harboring excessive numbers of dysregulated genes.

## Methods

### Sample collection

Sample collection was performed following approval of the Animal Care and Use Committees of the collecting institutions, and owners of all participating dogs signed an informed consent document. Five tumor samples were obtained through cystoscopy as directed by evaluation and treatment protocols decided upon following owner/oncologist consultation. The remaining tumor samples and adjacent normal tissues were collected during necropsy when the owners elected to have their dogs euthanized due to disease progression. Appropriate care was taken to collect tissues within 30 min of time-of-death. Tissues were histopathologically confirmed to be iUC. Upon collection, tissues were snap frozen and stored at − 80 °C until processed for analysis. Whole blood samples were collected in 3–6 ml EDTA or ACD tubes and stored at 4 °C prior to DNA extraction. Genomic DNA was extracted using established protocols [32]. DNA and RNA were extracted from flash frozen tissue samples using the AllPrep kit (Qiagen Corp., Alameda, CA). All samples were stripped of identifiers, numerically coded, and aliquoted for long-term storage at − 80 °C. Tumors were genotyped for the previously described BRAFV595E somatic mutation using amplicon resequencing and/or restriction fragment length polymorphism (RFLP) digestion as previously described [29].

### RNAseq

RNA samples from tumors and adjacent normal tissue samples were selected that exceeded a Bioanalyzer (Agilent, Santa Clara, CA) quality score of seven and quantified using a Qubit fluorometer (Qiagen, Alameda, CA). Unstranded RNA-Seq libraries were constructed from 1 μg total RNA using the Illumina TruSeq RNA Sample Prep Kit, v2 (Illumina, Inc., San Diego, CA). The resulting cDNA was fragmented using a Covaris E210 (Covaris, Inc., Woburn, MA). Library amplification was performed using 10 cycles to minimize the risk of over-amplification. Unique single-index barcode adapters were applied to each library. Libraries were pooled in equimolar ratio and sequenced together on an Illumina HiSeq 2500 with v4 flow cells and sequencing reagents (San Diego, CA). At least 37 million 127-base read pairs were generated for each individual library. Data was processed using RTA 1.18.61 and CASAVA 1.8.2 (Illumina, Inc., San Diego, CA). The RNAseq data is available in the NCBI short-read archive (SRA) under BioProject ID PRJNA559406.

Sequence reads were aligned to the CanFam3.1 reference using the STAR 2-part aligner [33], with genome resources generated from the CanFam3.1 fasta file downloaded from UCSC genome browser [34]. The initial guided alignment was performed using an Ensembl GTF reference file while documenting all splice junctions observed. The genome was regenerated including all known splice junctions and any novel junctions that occurred in a minimum of five reads. A second alignment was performed using the regenerated genome.

BRAFV595E mutation status was rechecked using base calls-per-read at the genomic position chr16:8296284. One matched normal sample that displayed mutant alleles at this position and one tumor that conflicted with original genotype results were excluded from further analysis. Because some matched normal tissues were used as controls, we verified the presence of distinct non-overlapping normal and tumor clusters using principal component analysis (PCA) and multidimensional scaling (MDS) of genome wide expression values before proceeding with differential expression analysis. The final sample set consisted of 15 tumors and five normal tissue samples of which three were from unaffected adjacent tissue and two were normal urothelial tissue. To perform a replication analysis we selected a comparable dataset of 12 tumors, eight with and four without BRAFV595E mutations, and four normal tissue samples available from previously published data [26]. Samples were not combined for a single analysis due to differences in RNAseq methodologies.

### Differential expression and clustering

HTSeq [35] was used to perform counts of all reads aligned to each of 32,704 genes annotated in Ensembl CanFam3.1.95, and for 13,173 predicted non-coding transcripts [36]. Genes that were not expressed were removed from the analysis leaving a final total of 32,938 predicted genes (24,509 Ensembl genes + 8429 predicted non-coding genes without Ensembl IDs). Both variance stabilizing and regularized log transformation was used to normalize counts for clustering by PCA and MDS. Clustering was performed both with and without normal samples and on the Ensembl and predicted non-coding genes, independently. ConsensusCluster was used to determine cluster membership based on stability of assignment after sub-sampling the data 1000 times [37].

Differential expression between tumors and normal tissues was calculated using DESeq2 [38]. A gene was considered significantly over or under expressed if the absolute value of the log2fold change was greater-than or equal-to one and the False Discovery Rate (FDR) adjusted *p*-value (q-value) was < 0.01. We chose a slightly higher than usual baseline for significance (*p* = .01 rather than .05) to increase confidence given small numbers of samples.

### Positional expression

Each gene was assigned a genomic position equal to the average of the positions centered between the first and last base in all annotated transcripts, as per Ensembl CanFam3.1.95 or CanFam3.1plus [36] The genome was divided into one Mb siding windows, overlapping by 750 Kb and gene counts within each segment were based on the assigned position. Using a hypergeometric distribution we calculated a *p*-value for over-representation of up- or down-regulated genes in each Mb window using the R script phyper. This was repeated for upregulated and down regulated genes, independently, in each dataset. For overlapping windows with significant *p*-values, the distances were summed, and p-values calculated for the entire region.

### Individual expression

To determine the significance of changes in expression level of each gene per individual, z-scores were calculated for each gene by comparison to the mean and standard deviation of the normal tissue expression level after variance stabilizing transformation, similar to that described previously [39]. A z-score of +/− 2.5 was required to indicate a significant change in expression. Transcripts per million (TPM) were also calculated per gene for each sample by correcting the read counts for the average coding length of the gene in each individual sequence [40].

### Gene-set enrichment and regulatory predictions

Approved symbols of genes that appeared dysregulated by at least two-fold, with a corrected *p*-value of less than 0.01, were compared to compiled gene lists indicated below to identify over-representation of any collated gene group in the tumor expression data. The systems used were the GSEA MutSig database version 6.2 [41, 42] which uses hypergeometric distribution to assess enrichment of genes from collated gene sets within a list of dysregulated genes, and Ingenuity Pathway Analysis (IPA) [43], which uses both the list of genes and their relative expression values to predict activity of upstream regulatory genes. The top-ten results by q-value are reported for the hallmark and curated gene sets (MutSig) and top-ten bias corrected z-scores with *p*-values <.05 for the upstream regulators (IPA).

## Results

### Sequencing coverage and QC

The complete transcriptomes of seven histologically confirmed canine InvTCC tumors carrying the BRAFV595E mutation (BRAFV595E), four tumors lacking the mutation (BRAFwt), and three matched normal tissues were sequenced to an average of 45.9 million reads per sample (range 39.4–60.7 million). These data were combined with the transcriptomes of four tumors and two normal tissue samples that were previously published [29], bringing the total number of samples to 15 tumors and five normal tissue samples. Clinical characteristics of this tumor set is described in Table 1.

**Table 1:** Characteristics of the dogs with bladder cancer from which tumor tissue was collected

Characteristic	Outcome
Age	Median 10 Years (Range 6–14 years)
Gender	7 Spayed Female
	6 Neutered Male
	2 Intact Male
Breed	4 Scottish Terriers
	3 Mixed Breed
	3 Beagles
	2 Shetland Sheepdogs, 1 West Highland White Terrier, 1 German Shorthaired Pointer, 1 Miniature Pinscher
TNM Stage at Tissue Collection	T2N0M0–5 Dogs
	T2N0M1–4 Dogs
	T2N1M1–2 Dogs
	T3N0M0–1 Dog
	T3N1M0–1 Dog
	T3N1M1–1 Dog
Prior Therapy	8 Dogs Had Prior Therapy Including: a Cyclooxygenase Inhibitor (7 Dogs), Leukeran (3 Dogs), Vinblastine-Folate Conjugate (2 Dogs), Vinblastine (1 Dog), Carboplatin (1 Dog), 5 Azacitidine (1 Dog), Intravesical Mitomycin C (1 Dog)
Tissue Collection Method	5 tumor tissues were collected by cystoscopy, 10 tumor tissues were collected at necropsy
Mutation Status of Tumor	11 tumors carried the BRAFV595E mutation, 4 tumors had no BRAF mutation

### Differential expression

**Fig. 1:**
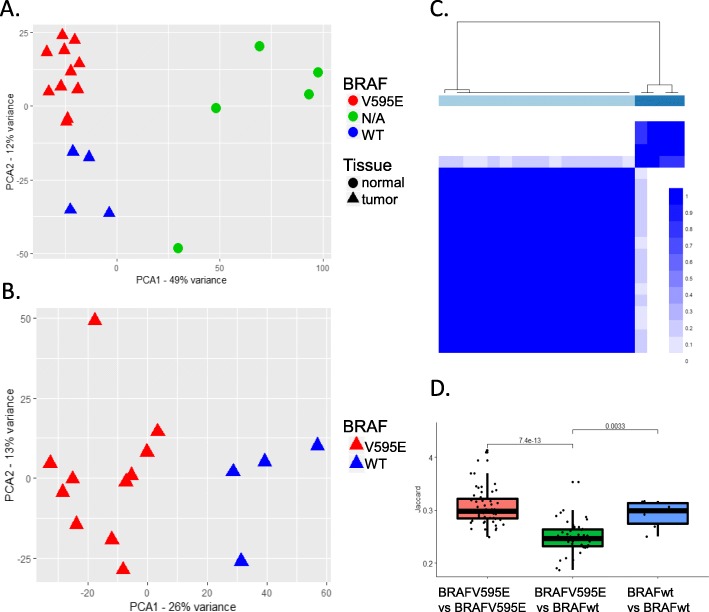
Canine iUC tumors cluster into two distinct groups. **a**) Principal component analysis divides normal from tumor tissues. **b**) After removing the normal tissues, the first principal component separates tumors with BRAFV595E mutations from those that do not carry the mutation. **c**) Consensus clustering based on expression data shows two clusters of tumors. Horizontal line under dendrogram: light blue = BRAFV595E tumors, dark blue = BRAFwt tumors. Heatmap blue shades from light to dark with increased confidence. **d**) Distribution of Jaccard similarity scores among BRAFV595E tumors (coral), BRAFwt tumors (light blue) and between the two mutation types (green)

Coverage was calculated per gene for a total of 45,877 genes, which includes both protein coding and noncoding genes such as long noncoding RNAs and antisense RNAs. Of those, 32,938 were expressed in the tumor and/or normal urothelial tissues. Comparing data from tumors to that from normal samples, 3587 genes annotated in Ensemble v1.95 were differentially expressed (DE), as were 778 predicted, non-coding genes (Additional Files 1 and 2). PCA and MDS clustering of both expression datasets separated normal versus tumor samples along the first dimension and tumors carrying the BRAFV595E mutation from the BRAFwt tumors along the second dimension (Fig. 1a). When normal samples were removed from the analysis, the tumors split into two distinct clusters comprised of those with the BRAFV595E mutation and those without (Fig. 1b). Consensus clustering assigned the tumors to each cluster with an item consensus score averaging > 0.99 (range 0.965 to 1.0) (Fig. 1c).

To confirm that expression signatures differed significantly between the BRAFV595E and BRAFwt tumors, gene expression was scored relative to normal tissues for each tumor sample independently. After assembling an individual list of genes for each sample with significant z-scores, Jaccard similarities were calculated for all tumor pairs. BRAFV595E tumors shared significantly more DE genes with each other than with BRAFwt tumors (pvalue = 7.4e-13). Similarly, BRAFwt tumors share significantly more DE genes with each other than with BRAFV595E tumors (pvalue = 0.0033) (Fig. 1d). This pattern is also observed using up- or down-regulated genes separately (Additional File 3: Supplementary Fig. 1).

**Fig. 2:**
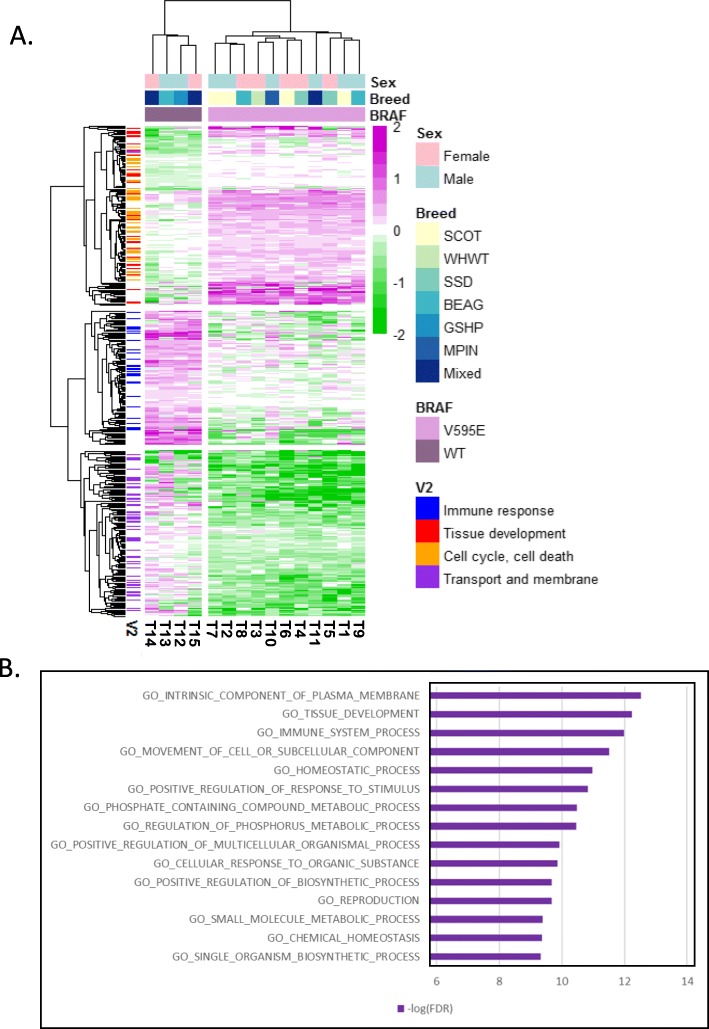
Expression profile of 398 genes separates the BRAFV595E tumors from BRAFwt tumors. **a**) Heatmap of canine iUC tumor expression from 398 genes that are oppositely expressed between the two tumor clusters. Tumor samples are clustered by Euclidean distance, genes are clustered by correlation. Expression levels are normalized compared to expression from normal tissues. **b**) Top 15 GO terms over-represented in the 398 genes

Following determination of significantly different expression patterns in BRAFV595E and BRAFwt tumors, differential expression analysis was performed for each group independently, comparing to normal tissue samples. The BRAFV595E cluster of tumors displayed 3839 DE genes, with a minimum two-fold increase or 0.5 reduction in expression (log2fold ≥1 or ≤ − 1) and Benjamini–Hochberg corrected *p*-values of < 0.01; 1576 up- and 2263 down-regulated. Using the same metrics, BRAFwt tumors were differentially expressed at 3724 genes; 1727 up- and 1997 down-regulated. Comparing the two tumor clusters, 2025 genes were similarly expressed in both, and only eight genes show significant expression in the opposite direction between the clusters. There are 398 genes that are significantly over- or under-expressed in one cluster with the reverse or unchanged expression (− 0.5 < log2fold change < 0.5) in the other. This minimal set of genes differentiates the two tumor clusters (Fig. 2a, Additional File 4).

### Overrepresentation of gene groups

The 398 genes form three clades with similar expression patterns within each tumor cluster. These clades largely comprise genes with four primary functions; tissue development and cell cycle/cell death (64/122 genes in gene clade 1), immune response (24/85 genes in gene clade 2) and plasma membrane and membrane transport (43/104 genes in gene clade 3). These functions are also the top three identified in the enrichment analysis of the 398 genes (Fig. 2b). The immune response gene clade is upregulated in BRAFwt tumors. Tissue development genes are upregulated in BRAFV595E tumors and down regulated in BRAFwt tumors. Cell cycle and cell death are upregulated in the BRAFV595E tumors, and the membrane associated genes are down-regulated (Fig. 2a). The genes from these functional groups comprise 42% (131) of the canine iUC tumor cluster identifying genes with unique human orthologs (Additional File 5).

To confirm our findings, we assessed the expression of the 131 genes from these top functional groupings in a dataset of RNAseq results from eight BRAFV595E tumors and four BRAFwt tumors published previously [26]. We were able to divide these data into two hierarchical clusters based on Euclidian distance, with eight of eight BRAFV595E tumors in one cluster and three of four BRAFwt tumors in a second cluster. The division of genotypes among the two clusters is significantly different than random (Fishers exact pvalue = 0.0182)(Additional File 3: Supplementary Fig. 2).

### Positional expression patterns

**Fig. 3:**
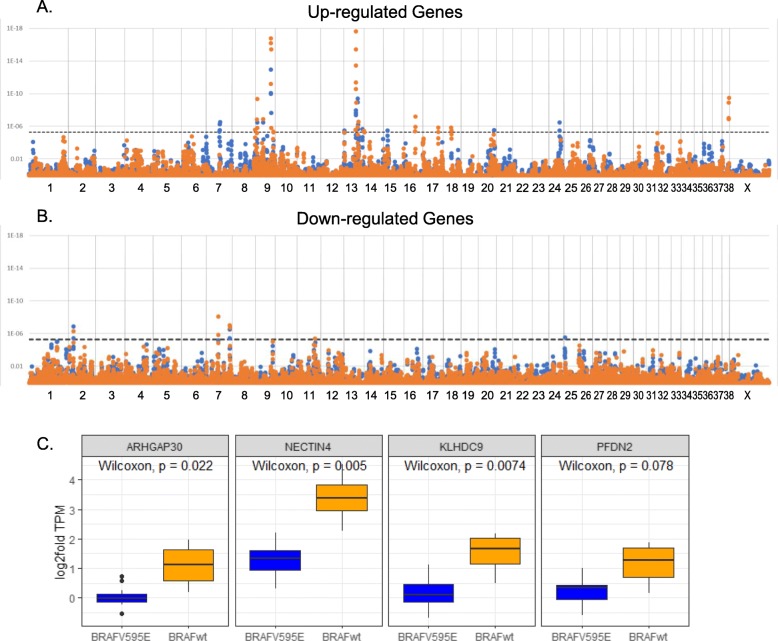
Positional analysis identifies loci on 15 chromosomes that contain significant numbers of dysregulated genes. The graphs are divided by **a**) upregulated genes and **b**) down regulated genes. The x-axis indicates the chromosomes from 1 to X. The y-axis showss the *p*-value for over-representation of dysregulated genes within a 1 Mb region surrounding each point on the graph. The dotted line indicates the Bonferroni corrected p-value of 0.05. BRAFwt tumors are shown in orange, BRAFV595E tumors are shown in blue. **c**) The core genes from the up-regulated locus on chr38, syntenic to the human 1q23.3 copy number variant, are overexpressed in canine iUC tumors that do not carry the BRAFV595E mutation. Boxplots display the transcripts per million compared to normal tissues from BRAFwt (orange) and BRAFV595E (blue) tumors

Assessing the expression of genes based on chromosomal position reveals 25 non-overlapping regions on 15 chromosomes. Each region is significantly over-represented by either up- or down-regulated genes in one or both canine iUC tumor clusters (Fig. 3a &b, Table 2). Two chromosomal regions show a lack of expression in both BRAFV595E and BRAFwt tumors, and five show increased expression in both tumor types. Ten loci are unique to the BRAFwt tumors (eight up- and two down-regulated), six are unique to the BRAFV595E tumors (five up- and one down-regulated). Thirteen of the 25 regions are syntenic to regions of common copy number variants in human tumors [44].

**Table 2:** Location of clusters of dysregulated genes across the canine genome

	BRAFV595E				BRAFwt				
Locus^a^	Up^b^	p-val	Dn^c^	pval	Up^b^	pval	Dn^c^	p-val	Human ortholog^d^
2:15250–16,500	0	ns	11	8.90E-08	0	ns	10	2.70E-07	10p11.22–23
7:37500–38,750	0	ns	9	1.70E-04	0	ns	11	6.70E-07	1q42.12–13
7:41500–42,500	14	4.30E-06	2	ns	10	3.20E-03	5	ns	1q21.3
7:42750–44,250	13	2.90E-06	4	ns	3	ns	1	ns	1q21.3
7:73250–74,500	0	ns	9	5.20E-08	0	ns	7	7.40E-06	18p11.23
7:73500–75,000	0	ns	7	3.70E-06	0	ns	9	6.80E-10	18p11.23
8:71500–72,500	8	1.50E-03	0	ns	12	2.70E-06	0	ns	14q32.33
9:4750–5750	15	3.50E-07	0	ns	19	4.40E-10	1	ns	17q25.1
9:24000–25,000	14	3.40E-07	1	ns	15	1.30E-07	0	ns	17q21.32
9:47500–49,250	25	1.40E-10	2	ns	33	2.00E-16	4	ns	9q34.3
9:55000–56,000	12	5.00E-05	2	ns	14	4.40E-06	2	ns	9q34.11
11:55500–56,500	0	ns	6	1.10E-03	0	ns	8	3.90E-06	9q22.33
13:1500–2500	7	3.20E-06	0	ns	7	5.50E-06	0	ns	8q22.2
13:36250–38,750	28	2.10E-09	4	ns	45	1.30E-21	1	ns	8q24.3
13:36750–38,250	27	2.90E-10	2	ns	42	8.40E-22	0	ns	8q24.3
13:43000–44,000	10	1.20E-09	0	ns	8	1.20E-06	0	ns	4p12
13:45250–46,250	7	2.90E-05	0	ns	9	2.80E-07	0	ns	4q12
13:57750–58,750	8	2.20E-06	0	ns	2	ns	0	ns	4q13.2
14:250–1250	3	ns	0	ns	10	3.70E-06	0	ns	1q42.13
15:12750–13,750	9	3.40E-06	1	ns	6	2.80E-03	2	ns	1p33
16:35500–37,000	1	ns	3	ns	10	2.20E-07	3	ns	8p22–23.1
17:48250–49,250	4	ns	2	ns	12	1.40E-06	3	ns	2p13.1
18:24750–26,000	8	4.50E-03	1	ns	13	3.30E-06	0	ns	11p15.5
20:49500–50,500	12	2.90E-06	4	ns	10	2.10E-04	5	ns	19p13.2
24:33500–34,750	8	2.20E-06	0	ns	3	9.20E-02	0	ns	20q13.12
25:3000–4000	0	ns	8	3.00E-06	0	ns	4	1.30E-02	13q13.3
38:20500–22,250	7	ns	6	ns	21	2.20E-08	5	ns	1q23.3

^a^The positions are given in kilobases. ^b^Up is the number of genes with increased expression. ^c^Dn is the number of genes with reduced expression. ^d^Human ortholog is the orthologous region in human genome build GRCh38/hg38. *ns* not significant

The single locus with the highest number of dysregulated genes is on canine chromosome 13 between 37 and 38 Mb, with 33 out of 83 genes upregulated in the BRAFwt tumors and 21 out of 83 upregulated in BRAFV595E tumors. This pattern of upregulation extends over 2.5 Mb region in BRAFwt tumors and encompasses a 1.5 Mb region in BRAFV595E tumors, yielding a total of 45 and 28 genes with increased expression, respectively (pvalue = 8.4e^− 22^ and 2.9e^− 10^). This region corresponds to the human chromosome 8q24.3, a locus commonly amplified in multiple human tumor types, including urothelial cancer [44–46].

One of the loci upregulated specifically in BRAFwt tumors spans 20.50 to 22.24 Mb on canine chr38, a region syntenic to human chromosome 1q23.3. The region contains 21 upregulated genes. Chromosome 1q23.3 is amplified in > 50% of human iUCs and has been associated with increased tumor stage and grade [47] as well as decreased survival time [48]. Of the nine genes that comprise the core of the human amplification region [44], seven are upregulated in BRAFwt tumors compared to normal tissue and display significantly higher expression than BRAFV595E tumors (Fig. 3c and Additional File 3: Supplementary Fig. 3). *Nectin4*, which is commonly used as a marker for amplification of the human 1q23.3 locus demonstrates the highest levels of expression in the BRAFwt tumors compared to normal tissue samples (log2fold = 5.89, adjP = 4.09e^− 10^). Nectin-4 protein is a cell surface adhesion molecule previously named poliovirus receptor-related 4 (PVRL4). The canine protein is 94% identical to the human, which is highly expressed in 60% of human bladder tumors [49].

## Discussion

Previous studies have established the dog as a strong model for clinical, pharmacologic and genetic studies of human iUC. To improve the clinical utility of the model we sought to develop a better understanding of the genomic profile associated with canine iUC by identifying differentially expressed genes among canine tumors versus normal tissue. We, and others, had previously defined a mutation in the *BRAF* gene and encoded protein in over 80% of canine iUC (BRAFV595E), that corresponds to the common BRAFV600E variant observed in humans [29, 30]. This is in contrast to human iUC for which the same mutation is rare. It is, however, common in human metastatic melanoma, colon and thyroid cancer, and rare leukemias [50–53].

A total of 3588 differentially expressed genes were identified by RNASeq in 15 iUC canine tumors compared to five normal tissue samples. We analyzed the top 100 up- and down-regulated genes from two previous studies of seven and eleven canine iUC tumors, and identified 65 and 81% of the same genes, respectively [27, 28]. Dividing our data by tumor cluster, the top 100 genes from past studies are most similar to the results we observe in the BRAFV595E tumors; 67 and 83% identical dysregulated genes, respectively. In contrast, the BRAFwt cluster shares only 49 and 62% of dysregulated genes. This is likely due to the overabundance (up to 87%) of BRAFV595E mutations in canine iUC [29, 30], thus skewing all lumped results toward the pattern of BRAFV595E tumors.

A total of 178 DE genes appear in all three studies, ignoring BRAFV595E mutation status. Of these (Additional File 6), 29 are associated with activation of *EGFR* and/or *FGFR1* gene products in varying types of cancer (FDR qvalue = 4.68e^− 15^ and 3.74e^− 15^, MutsigDB). *EGFR* and *FGFR1* appear to work together to increase tumorgenicity in lung cancer, and active *FGFR1* can increase resistance to EGFR targeted therapies [54]. In this study we observe that *ERBB2* is overexpressed in all canine iUC tumors. Five of those had significant z-scores (z-score = 2.54–4.65, equivalent to *p* < 0.01), and the remainder had z-scores surpassing standard measures of significance (z-score- = 1.70–2.49, equivalent to *p* < 0.05). HER2, the gene product of *ERBB2* is upregulated in 37% of human iUCs and, combined with *EGFR*, is associated with advanced disease stage at diagnosis and increased risk of recurrence [55]. HER2 overexpression has been identified by immunohistochemistry in 57% of canine iUCs [56] and is predicted to be an upstream regulator of gene expression in canine iUCs [28]. Some growth factors, including *EGFR* and *ERBB2*, are the target of directed therapies for the treatment of iUCs in humans [57]. These findings suggest that canine iUC is an excellent platform for expanding our foundational understanding of growth factor receptor-targeted therapies. Clinical *EGFR* inhibitor trials are currently underway in dogs [3, 58].

We did not observe clustering based on luminal or basal signatures. Rather, all tumors in this dataset appear to have a luminal-like expression signature based on gene sets developed to distinguish these two molecular subtypes (Additional File 3; Supplementary Figs. 4A and B) [15, 26]. Our gene enrichment analysis demonstrates that all tumors in this study have an excess of dysregulated genes associated with human luminal-type tumors (pvalues = 7e^− 83^ and 3e^− 125^)(Additional File 7). Although not evident from the enrichment analysis, six tumors from the BRAFV595E cluster show upregulation of genes that are commonly used to identify the basal expression signature in human tumors in addition to luminal-type tumor marker genes (Additional File 3; Supplementary Fig. 4C). This expression pattern might indicate a small mixed tumor cluster, similar to the one termed UroB by Sjodahl et al. [17] UroB tumors can show signatures of both luminal and basal subtypes and have been assigned to both tumor types [17].

In the dataset presented here the tumor clusters correlate perfectly with the presence or absence of the BRAFV595E mutation. It is not uncommon for a somatic mutation to be overrepresented in a single expression cluster. For instance, *FGFR3* mutations are found primarily in the luminal-papillary cluster of human iUC tumors [20]. However, perfect concordance is unusual given tumor heterogeneity. The exact concordance observed in this study is likely due to small numbers of tumors, and future analyses may reveal an overrepresentation of the BRAFV595E mutation in a single canine tumor cluster, much as is observed human studies. It is possible that non-conforming tumors may represent early stage tumor development in which the BRAFV595E mutation is not yet prevalent, or they may harbor unidentified somatic changes that activate the same pathways. Further investigation of BRAFwt tumors may reveal early mutations that initiate tumorigenesis, making them particularly good targets for therapy.

In this study, we included tumors from several dog breeds with an established increased risk of iUC. Nine out of the eleven BRAFV595E tumors were from four (Scottish Terrier, West Highland White Terrier, Shetland Sheepdog and Beagle) of the top seven high-risk breeds [4]. Only one of the samples in the BRAFwt cluster was from a high-risk breed. While the increased risk suggests a genetic predisposition to iUC, this is the first data to suggest that there may be common inherited mutations which may contribute to somatic mutation and expression patterns.

Analysis of the BRAFwt tumors shows an increase in immune activity, with 24 immune system-associated genes significantly upregulated compared to observations in both normal tissues as well as BRAFV595E tumors. This includes statistically significant enrichment of genes associated with interferon response and cytokine signaling pathways, and multiple upstream cytokine regulators (Fig. 2, Additional Files 5 and 8). Immune system gene signatures in human bladder tumors are considered to be associated with the infiltration of immune cells, i.e., not tumor cells [14, 17, 20]. Human tumors with strong evidence of immune infiltration have responded better to immune checkpoint therapies in some clinical trials [59]. Additionally, luminal infiltrated tumors respond well to radiation therapy, possibly because radiation triggers an immune response [60]. *CCR5*, one of the immune related genes upregulated in BRAFwt tumors is also upregulated in some human mammary tumors and appears to promote metastasis [61]. Antagonists of *CCR5* are currently being assessed for their anti-tumor activity in aggressive tumors that express the gene [61–63].

Positional analysis of expression data highlights syntenic human genome regions that harbor common somatic copy number variants important in tumor development. Ten chromosomal regions in our study correspond to sites of recurring copy number amplification in a study of 11 different human tumor types, including bladder cancer [44]. Four of these occur on canine chromosome 13. Fluorescence in situ hybridization analysis reveals amplification of the entirety of chromosome 13 in canine iUCs [1], as does the expression level of individual genes in the region [27]. Our results highlight five regions on chromosome 13. The largest is 2.5 Mbs in the BRAFwt tumors and 1.5 Mbs in BRAFV595E (pvalue = 1^− 21^ and 3^− 10^ respectively). This region corresponds to human chromosome 8q24.3. This locus is amplified frequently in multiple human tumor types including those of the bladder and prostate [46, 64] Lymphocyte antigen 6 family member K (*LY6K*) is a gene associated with cell growth and is upregulated in human cell lines displaying 8q24.3 amplification [46]. In canine tumors, we observe that *LY6E* is amplified in BRAFwt tumors but in only half of BRAFV595E tumors. Genes at 8q24 have also been linked to *MYC* activity, which is located 10Mbs upstream of the amplified region. There is no significant change in expression of *MYC* between canine tumors and normal tissues in either cluster. However, pathway analysis predicts that *MYC* is activated as an upstream regulator of transcription in the BRAFV595E tumors (activation z-score = 4.4, *p* = 8.3e-6) but not in the BRAFwt tumors. These findings suggest there are different functional outcomes related to amplification at the 8q24.3 locus within canine iUCs. Thus, a comparison of canine and human syntenic regions could help define the functional and disease-relevant variants in loci involving large structural variants.

Of the four additional regions that display significantly upregulated groups of genes on canine chromosome 13 three are frequently amplified in human tumors. The first is located at 1.5 to 2.5 Mb and corresponds to human 8q22.2, which is duplicated in human bladder cancers [65, 66], while the second and third regions, from 43 to 44 Mb and 45.2 to 46.2 Mb, correspond to human 4q12, which is amplified in numerous human tumor types. The last locus harbors a cluster of over-expressed genes located between 57.7 and 58.7 Mb. Genes showing increased expression in this region include three different transmembrane protease serine 11 genes (*TMPRSS11d, e and g*). *TMPRSS11e* is associated with decreased survival in iUC patients [67, 68] and has been identified as a primary hub gene in co-expression networks marking iUC tumor progression [69]. This family of transmembrane proteins has not been extensively studied in iUC and, based on these results, is worth further investigation.

Our data demonstrates that the *nectin4* gene is highly upregulated in BRAFwt tumors. *Nectin4* lies in a region syntenic to human chromosome 1q23.3, and is overexpressed in human bladder and breast tumors, highlighting it’s potential as a drug target for epithelial cancers [49, 70]. A phase 1 clinical trial in humans has accomplished a 40% response rate with an antibody-drug conjugate targeting nectin4, and phase two and three trials are underway [71]. Although we predict that the entire locus is amplified in BRAFwt tumors, *nectin4* is expressed above expected levels in all of the canine iUC tumors analyzed here, highlighting yet another clinical pathway in which studies of canine iUC could play a role in human treatment development.

## Conclusions

In this study we examined expression patterns in BRAFV595E and BRAFwt tumors compared to normal tissue samples. We find distinct patters of dysregulated genes, with clusters of over and under-expressed genes limited to a small number of the dog’s 38 chromosomes. Notably, many of these regions highlight syntenic regions in the human genome that are associated with initiation or progression of human tumors, as well as long term and often deleterious outcomes. Future studies that include whole genome sequencing of large numbers of tumor/normal pairs will permit analysis of genotype/phenotype correlations.

The domestic dog is increasingly under consideration as a genetic system for the study of human disorders with underlying genetic components. In this study we further enhance that claim, demonstrating many molecular similarities in expression between human and dog iUCs. Our identification of these two tumor types in canine iUC has allowed us to identify new similarities between human and canine tumors and validate previously established observations. The fact that > 85% of canine tumors harbor BRAFV595E mutations which are correlated with expression patterns provides us with a mechanism to parse canine tumors in a way that is absent in humans, permitting early sub set analysis and improved matching for clinical trials of tumors with likely differing clinical outcomes. Further, our demonstration of expression profiles highly associated with somatic genotypes outlines distinct avenues to be explored for furthering our understanding of the underlying tumor biology of iUCs.

## Supplementary information

**Additional file 1:.** Figures S1–S4. Supporting figures for results.

**Additional file 2:.** Table S1. Expression values of Ensembl genes.

**Additional file 3:.** Table S2. Expression values of predicted non-coding genes.

**Additional file 4:.** Table S3. Genes that are differentially expressed between BRAFV595E and BRAFwt tumors.

**Additional file 5:.** Table S4. Gene groups overrepresented in the genes that distinguish BRAFV595E tumors from BRAFwt tumors.

**Additional file 6:.** Table S5. Comparison of gene expression values from previously published datasets.

**Additional file 7:.** Table S6. Curated gene sets over-represented in canine iUC.

**Additional file 8:.** Table S7. Hallmark gene sets over-represented in canine iUC.

## Data Availability

The dataset supporting the conclusions of this article is available in the NCBI short-read archive (SRA) under BioProject ID PRJNA559406 and PRJNA308949. https://www.ncbi.nlm.nih.gov/sra

## Abbreviations

iUC: invasive urothelial carcinoma
RFLP: Restriction fragment length polymorphism
TPM: Transcripts per million
SRA: Short read archive
MDS: Multidimensional scaling
PCA: Principal component analysis
FDR: False discovery rate
DE: Differentially expressed
Mbs: Megabases
