# Supplementary material for: RNAseq expression patterns of canine invasive urothelial carcinoma reveal two distinct tumor clusters and shared regions of dysregulation with human bladder tumors
Source: BMC Cancer. 2020 Mar 24;20:251. doi: 10.1186/s12885-020-06737-0 (PMC7092566; doi:10.1186/s12885-020-06737-0)
Supplement: Supplementary file 3 — Additional file 3:. Table S2. Expression values of predicted non-coding genes. [file 12885_2020_6737_MOESM3_ESM.pptx]

## Slide 1
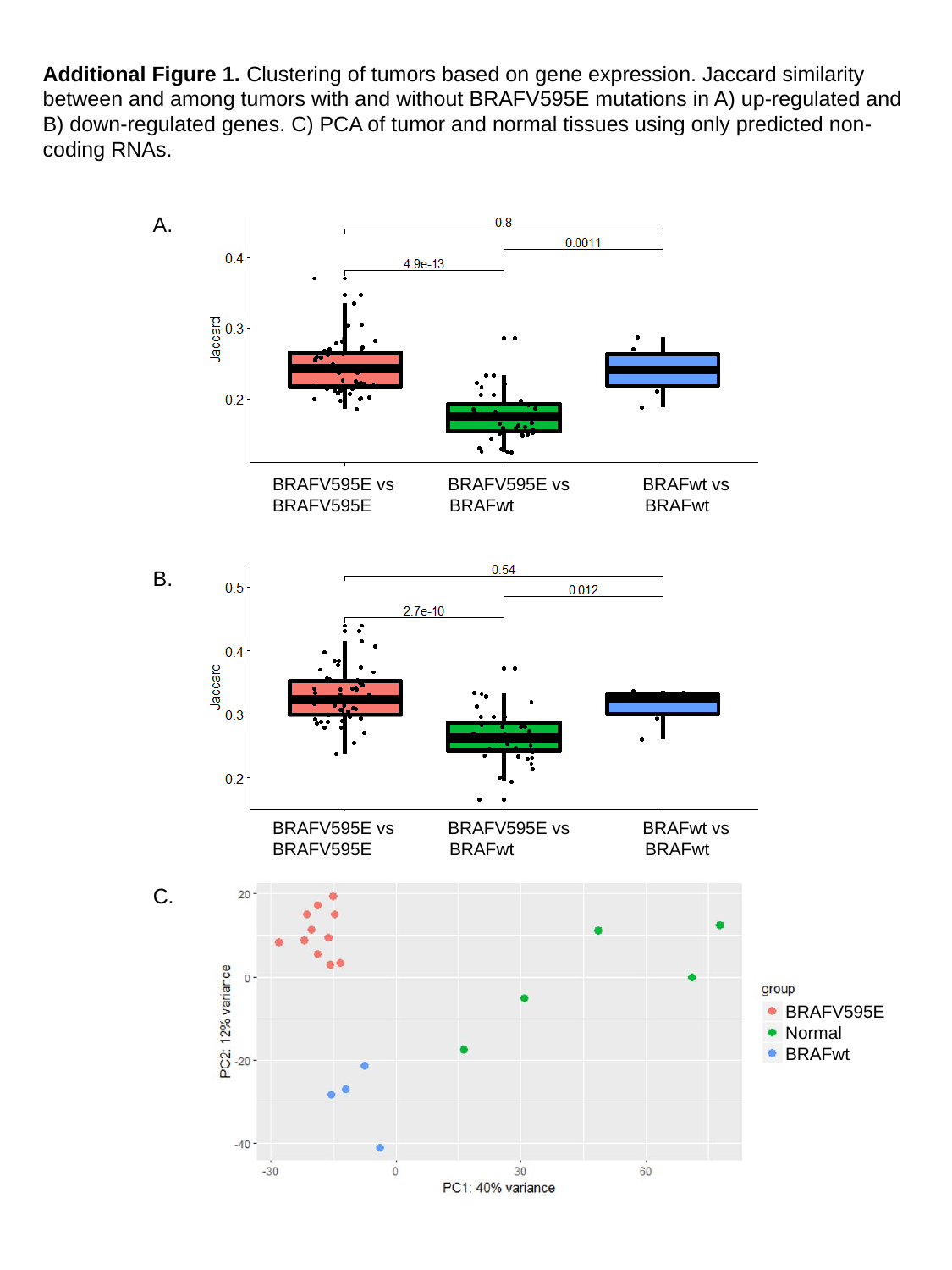

Additional Figure 1. Clustering of tumors based on gene expression. Jaccard similarity between and among tumors with and without BRAFV595E mutations in A) up-regulated and B) down-regulated genes. C) PCA of tumor and normal tissues using only predicted non-coding RNAs.
A.
BRAFV595E vs BRAFV595E vs BRAFwt vs
BRAFV595E BRAFwt BRAFwt
B.
BRAFV595E vs BRAFV595E vs BRAFwt vs
BRAFV595E BRAFwt BRAFwt
C.
BRAFV595E
Normal
BRAFwt

## Slide 2
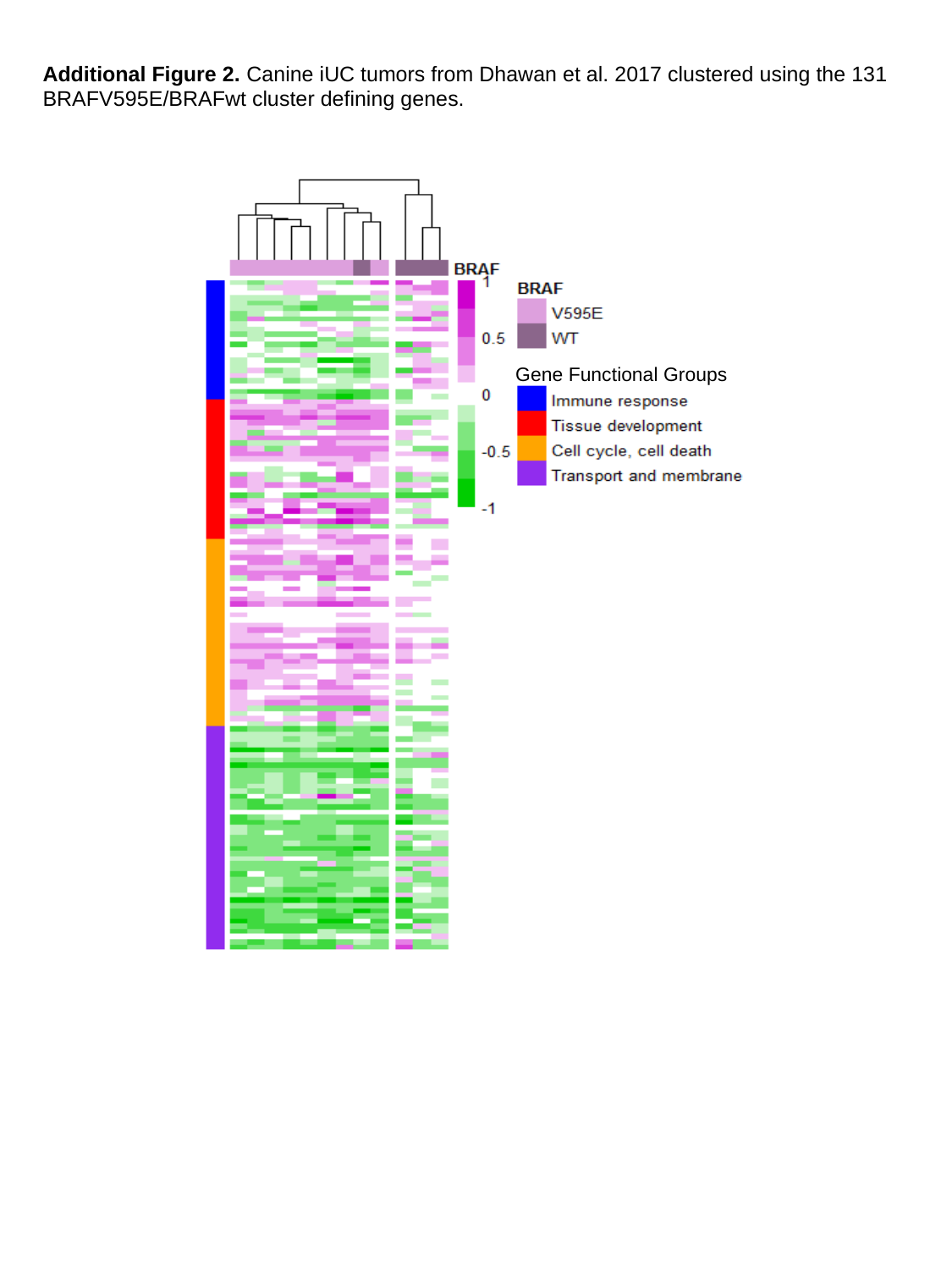

Additional Figure 2. Canine iUC tumors from Dhawan et al. 2017 clustered using the 131 BRAFV595E/BRAFwt cluster defining genes.
Gene Functional Groups

## Slide 3
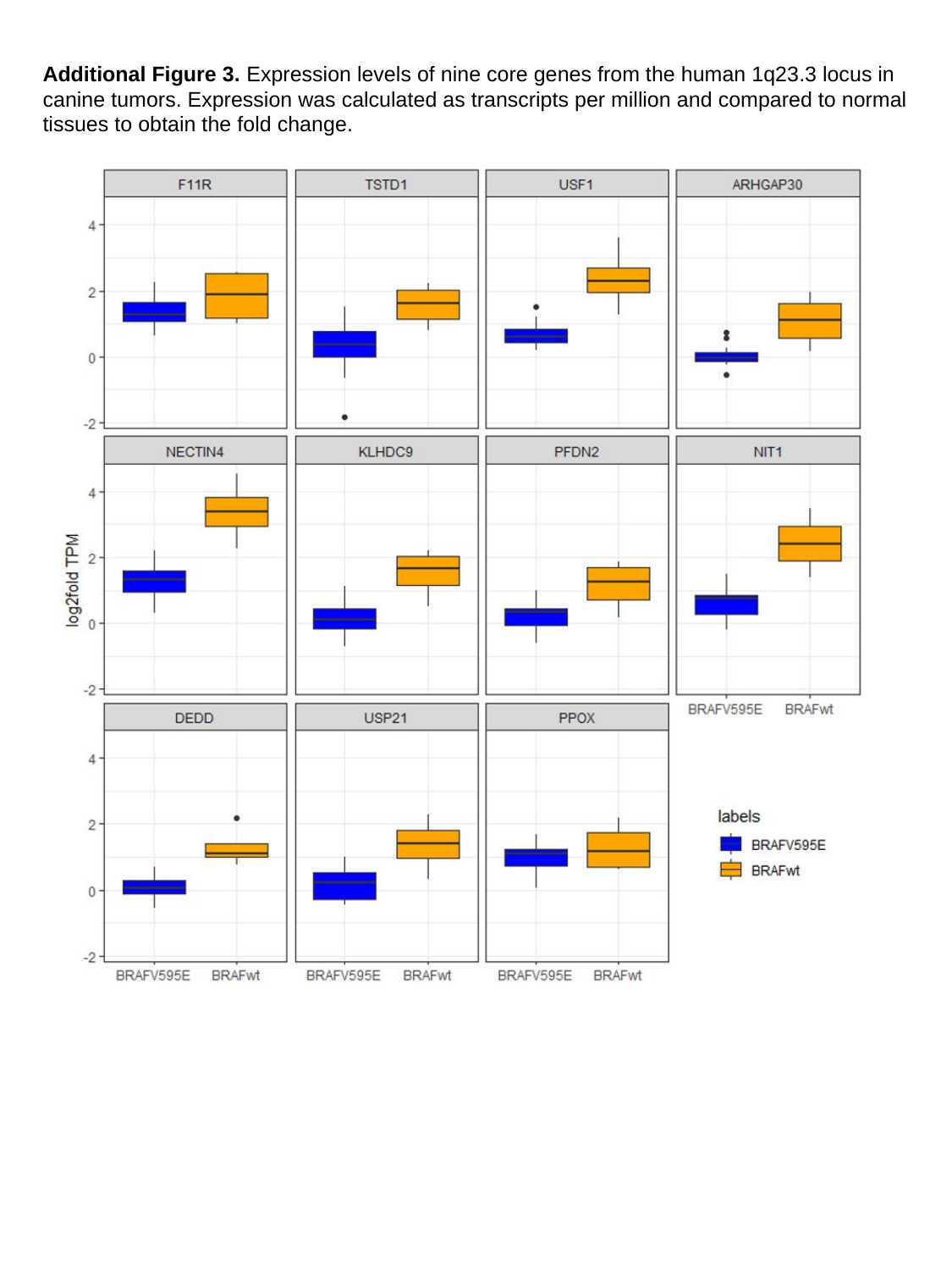

Additional Figure 3. Expression levels of nine core genes from the human 1q23.3 locus in canine tumors. Expression was calculated as transcripts per million and compared to normal tissues to obtain the fold change.

## Slide 4
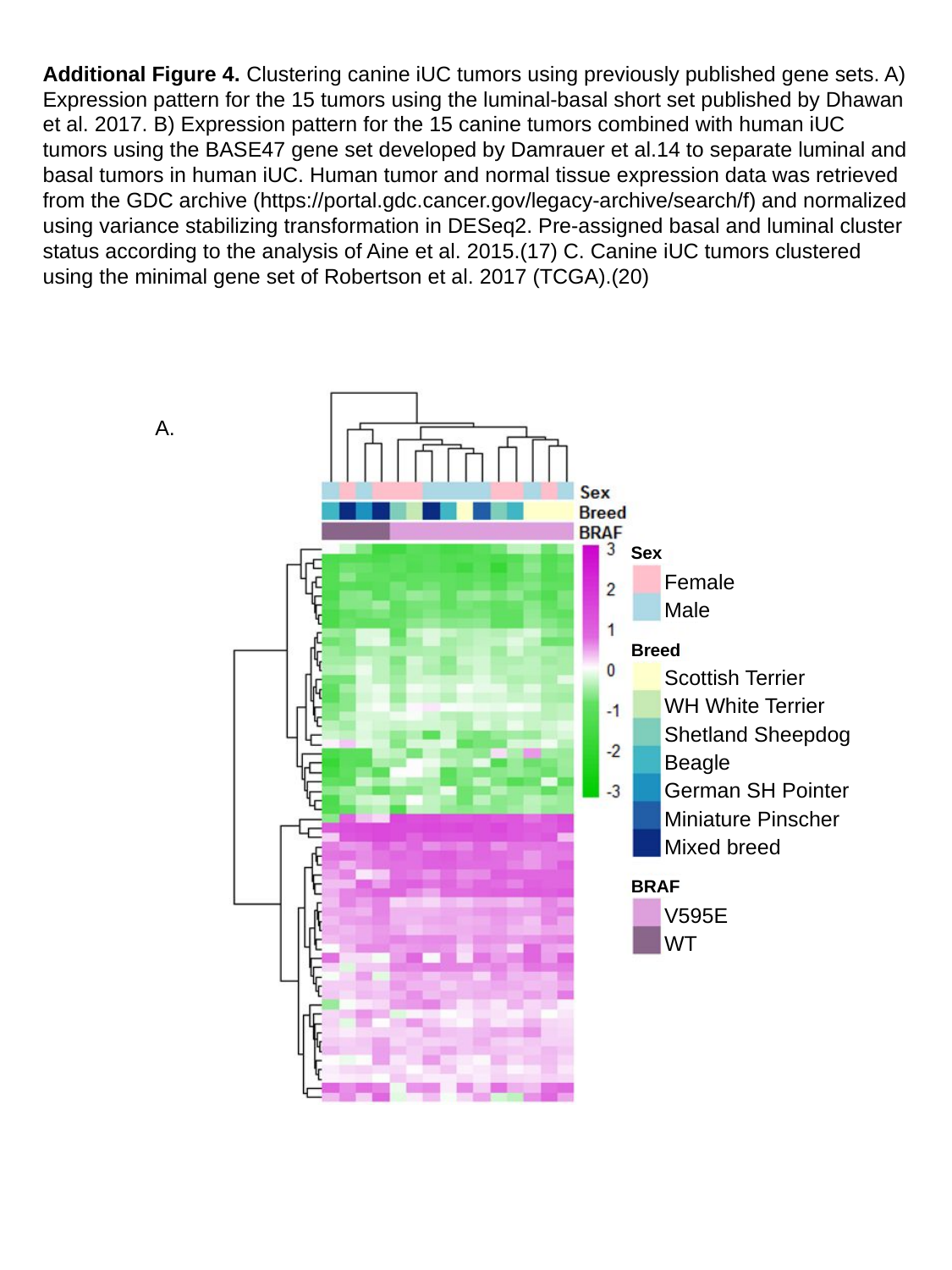

Additional Figure 4. Clustering canine iUC tumors using previously published gene sets. A) Expression pattern for the 15 tumors using the luminal-basal short set published by Dhawan et al. 2017. B) Expression pattern for the 15 canine tumors combined with human iUC tumors using the BASE47 gene set developed by Damrauer et al.14 to separate luminal and basal tumors in human iUC. Human tumor and normal tissue expression data was retrieved from the GDC archive (https://portal.gdc.cancer.gov/legacy-archive/search/f) and normalized using variance stabilizing transformation in DESeq2. Pre-assigned basal and luminal cluster status according to the analysis of Aine et al. 2015.(17) C. Canine iUC tumors clustered using the minimal gene set of Robertson et al. 2017 (TCGA).(20)
A.
Sex
Female
Male
Breed
Scottish Terrier
WH White Terrier
Shetland Sheepdog
Beagle
German SH Pointer
Miniature Pinscher
Mixed breed
BRAF
V595E
WT

## Slide 5
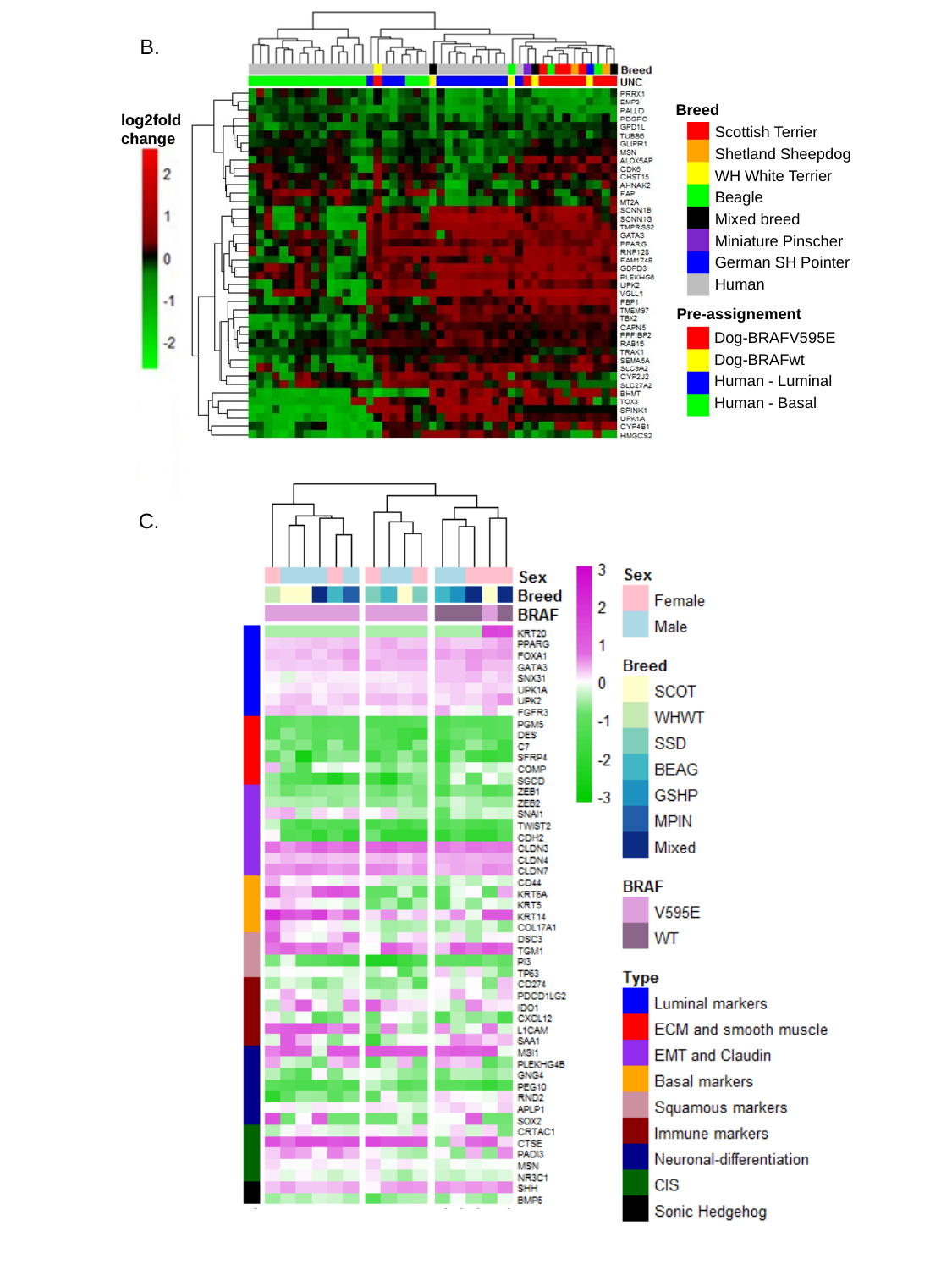

B.
Breed
Breed
log2fold
change
Scottish Terrier
Shetland Sheepdog
WH White Terrier
Beagle
Mixed breed
Miniature Pinscher
German SH Pointer
Human
UNC
Pre-assignement
Dog-BRAFV595E
Dog-BRAFwt
Human - Luminal
Human - Basal
C.
